# The phenomenology of dysphoric mood: exploring the lived experience

**DOI:** 10.1192/j.eurpsy.2025.180

**Published:** 2025-08-26

**Authors:** O. Doerr-Zegers, H. Pelegrina Cetran

**Affiliations:** 1 Facultad de Medicina Universidad de Chile, Hospital-Instituto Psiquiátrico, Santiago, Chile; 2 Universidad Católica Argentina, Mendoza, Argentina

## Abstract

**Abstract:**

Dysphoria is a complex phenomenon, which must be defined in the framework of different forms of affections. It belongs to the broader field of emotions, which are characterized by some essential features: i.e. movement, passiveness, transitoriness, and reference to the others. All these four essential features of emotion are specifically altered in depression. In discussing dysphoria, a first distinction is made between particular and global affections. The first type encompasses emotions and feelings, while the second one includes humor, mood and temper. Dysphoria belongs to one of these global affective states: the humor, which has to do with the spatial dimension of existence. In dysphoria, the patient experiences the world as oppressive and invasive of his/her intimacy; the others are lived as persons demanding answers or actions he/she is not able to fulfill. Finally, the phenomenology of dysphoria is analyzed through the four essential features described above and examples are given. Irritability – as Kraepelin taught us more than 100 years ago- is the most frequent psychic condition of these patients. The humor is very instable and depending, above all, on the interpersonal relationships. It is a typical humor of premenstrual disorder, but also of the borderline personalities, as Stanghellini and Rosfort (2013) have so clearly showed. We have also observed dysphoria in two other conditions, depression and mania, though with different nuances in the form of presentation. Finally, we would like to show how the different features characterizing emotion in general, which are derived from the etymology of the word, are present in dysphoria. First, the movement appears in dysphoria as a corporal restlessness always accompanying irritability. The dysphoric state is the opposite of being in peace with oneself and with the world. It is being under pressure, urgency, loss of control, impulsivity. Second, passivity: the subject cannot decide to be in one state or the other: dysphoria happens without notice and invades the subject, who is unable to defend himself against it. Borderline patients are unable to take distance from their emotional states and particularly from dysphoria, blaming others for the consequent discomfort. The third element is transitoriness. This feature is particularly observed in premenstrual disorder. Menstruation begins and the state disappears. Something similar occurs with pre-depressive dysphoria. In manias, by contrast, dysphoria only diminishes with the treatment. The last element we infer from the etymology of emotion is commotion, that is, its permanent reference to the other. The active participation of the other in the dynamic of dysphoria is particularly evident in borderline personalities, but also in depressive and manic dysphoria, but not in premenstrual syndrome.

**Disclosure of Interest:**

None Declared

